# Bis(1*H*-benzimidazole-κ*N* ^3^)bis­[2-(naphthalen-1-yl)acetato-κ^2^ *O*,*O*′]nickel(II) monohydrate

**DOI:** 10.1107/S1600536812010021

**Published:** 2012-03-14

**Authors:** Fu-Jun Yin, Li-Jun Han, Zhao Hong, Xing-You Xu, Li Ren

**Affiliations:** a Jiangsu Marine Resources Development Research Institute, Huaihai Institute of Technology, Lianyungang 222005, People’s Republic of China; b Department of Mathematics and Science, Huaihai Institute of Technology, Lianyungang 222005, People’s Republic of China; c Department of Chemical Engineering, Huaihai Institute of Technology, Lianyungang 222005, People’s Republic of China; d Huaiyin Insititute of Technology, Huaiyin 223003, People’s Republic of China; e Qian’an College, Hebei United University, Tangshan 063009, People’s Republic of China

## Abstract

In the title compound, [Ni(C_12_H_9_O_2_)_2_(C_7_H_6_N_2_)_2_]·H_2_O, The Ni^II^ cation is located on a twofold rotation axis and is six-coordinated in a distorted NiN_2_O_4_ octa­hedral geometry. The asymmetric unit consists of a nickel(II) ion, one 2-(naphthalen-1-yl)acetate anion, a neutral benzotriazole ligand and one half of a lattice water mol­ecule. The crystal packing is stabilized by O—H⋯O and N—H⋯O hydrogen bonds. The title compound is isotypic with its Cd^II^ analogue.

## Related literature

For the crystal structures of related 2-(naphthalen-1-yl)acetate complexes, see: Yin *et al.* (2011*a* ▶,*b* ▶); Liu *et al.* (2007 ▶); Yang *et al.* (2008 ▶); Tang *et al.* (2006 ▶); Ji *et al.* (2011 ▶). For the isotypic Cd^II^ complex, see: Duan *et al.* (2007 ▶).
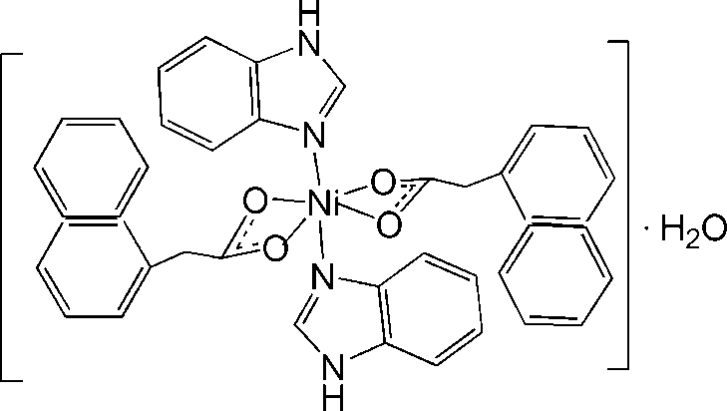


## Experimental

### 

#### Crystal data

[Ni(C_12_H_9_O_2_)_2_(C_7_H_6_N_2_)_2_]·H_2_O
*M* *_r_* = 683.37
Monoclinic, 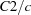

*a* = 11.573 (4) Å
*b* = 19.991 (7) Å
*c* = 14.290 (5) Å
β = 105.903 (4)°
*V* = 3179.5 (19) Å^3^
*Z* = 4
Mo *K*α radiation
μ = 0.66 mm^−1^
*T* = 298 K
0.10 × 0.10 × 0.10 mm

#### Data collection

Bruker APEXII CCD diffractometer
Absorption correction: multi-scan (*SADABS*; Sheldrick, 1996 ▶) *T*_min_ = 0.952, *T*_max_ = 0.952
11840 measured reflections
2807 independent reflections
1849 reflections with *I* > 2σ(*I*)
*R*_int_ = 0.083

#### Refinement

*R*[*F* ^2^ > 2σ(*F* ^2^)] = 0.050
*wR*(*F* ^2^) = 0.122
*S* = 0.99
2807 reflections
221 parameters
H atoms treated by a mixture of independent and constrained refinement
Δρ_max_ = 0.41 e Å^−3^
Δρ_min_ = −0.43 e Å^−3^

### 

Data collection: *APEX2* (Bruker, 2008 ▶); cell refinement: *SAINT* (Bruker, 2008 ▶); data reduction: *SAINT*; program(s) used to solve structure: *SHELXS97* (Sheldrick, 2008 ▶); program(s) used to refine structure: *SHELXL97* (Sheldrick, 2008 ▶); molecular graphics: *SHELXTL* (Sheldrick, 2008 ▶); software used to prepare material for publication: *SHELXTL*.

## Supplementary Material

Crystal structure: contains datablock(s) I, global. DOI: 10.1107/S1600536812010021/bg2448sup1.cif

Structure factors: contains datablock(s) I. DOI: 10.1107/S1600536812010021/bg2448Isup2.hkl

Additional supplementary materials:  crystallographic information; 3D view; checkCIF report

## Figures and Tables

**Table 1:** Hydrogen-bond geometry (Å, °)

*D*—H⋯*A*	*D*—H	H⋯*A*	*D*⋯*A*	*D*—H⋯*A*
O1*W*—H1*A*⋯O4	0.90 (8)	2.24 (8)	2.988 (6)	141 (8)
N2—H2⋯O4^i^	0.86	2.00	2.791 (4)	152

Symmetry code: (i) 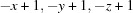.

**Fig. 1.**
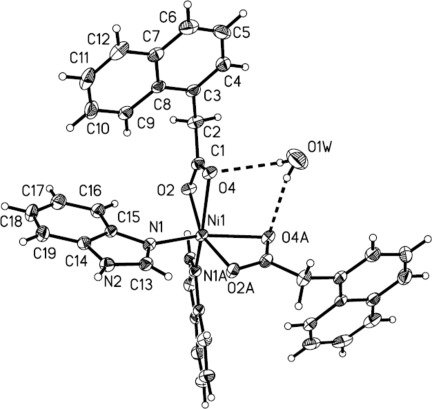
Molecule and the asymmetric unit of the title compound. Displacement ellipsoids are drawn at the 30% probability level.Hydrogenbonding is shown as dashed lines[Symmetry code: (A) 1-x, y, 1.5-z].

**Fig. 2.**
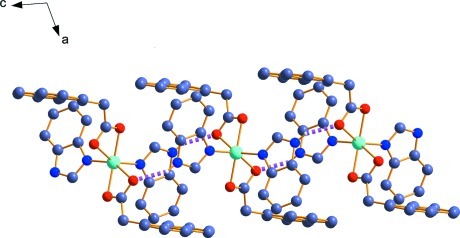
Part of the one-dimensional chain structure of (I) formed by N—H···O hydrogen bonds (dashed lines)along [001].

## supplementary crystallographic information

### Comment

In recent years particular interest has been devoted to the 1-naphthylacetate ligand in coordination chemistry due to its ability to form metal complexes (Yin *et al.*, 2011*a*, 2011*b*; Liu *et al.*,2007; Yang *et al.*, 2008; Tang *et al.*,2006 ; Ji *et al.*, 2011). The crystal structure of the title compound was determined as part of an ongoing study of the properties of nickel complexes containing imidazole ligands. In the title mononuclear metal complex, the asymmetric unit consists of a nickel cation, one 2-(naphthalen-1-yl)acetate anion,a benzotriazole ligand, and half of a lattic water molecule. The Ni^II^ cation is located on a two fold rotation axis and is six coordinated by two N from two benzotriazoles and four O atoms from two different 2-(naphthalen-1-yl)acetate anions, displaying a distorted NiN2O4 octahedral geometry, with Ni-O bond lengths in the range 2.056 (2)-2.288 (2)Å; the Ni-N bond length is 2.020 (3)Å. The crystal packing is stabilized by intermolecular N—H···O hydrogen bonding (Fig. 2) interactions which give rise to a one-dimensional chain structure. An isotypic cadmium(II) structure has been reported previously (Duan *et al.*, 2007).

### Experimental

The title compound was synthesized by the reaction ofNi(NO_3_)_2_.6H_2_O, (87.24 mg, 0.3 mmol), 1-naphthylacetic acid(93 mg, 0.5 mmol), benzotriazole (35.4mg, 0.3 mmol) and NaOH (20 mg, 0.5 mmol) in 16 mL of a water-ethanol (2:1) mixture under solvothermal conditions. The mixture was homogenized and transferred into a sealed Teflon-lined solvothermal bomb (volume: 25 ml) and heated to 160°C for three days. After cooling colorless the crystals of the title compound were obtained, which were washed with distilled water and absolute ethanol.

### Refinement

H atoms were placed in calculated positions, with N–H = 0.86 Å ; C—H = 0.93 Å or C—H = 0.97 Å and refined with a riding model, with *U*_iso_(H) = 1.2*U*_eq_(C).Water H atoms were located in Fourier difference maps and refined isotropically..

### Crystal data

**Table:**

[Ni(C_12_H_9_O_2_)_2_(C_7_H_6_N_2_)_2_]·H_2_O	*F*(000) = 1424
*M**_r_* = 683.37	*D*_x_ = 1.428 Mg m^−^^3^
Monoclinic, *C*2/*c*	Mo *K*α radiation, λ = 0.71073 Å
Hall symbol: -C 2yc	Cell parameters from 1368 reflections
*a* = 11.573 (4) Å	θ = 3.5–23.4°
*b* = 19.991 (7) Å	µ = 0.66 mm^−^^1^
*c* = 14.290 (5) Å	*T* = 298 K
β = 105.903 (4)°	Block, colourless
*V* = 3179.5 (19) Å^3^	0.10 × 0.10 × 0.10 mm
*Z* = 4	

### Data collection

**Table:**

Bruker APEXII CCD diffractometer	2807 independent reflections
Radiation source: fine-focus sealed tube	1849 reflections with *I* > 2σ(*I*)
Graphite monochromator	*R*_int_ = 0.083
φ and ω scans	θ_max_ = 25.0°, θ_min_ = 2.1°
Absorption correction: multi-scan (*SADABS*; Sheldrick, 1996)	*h* = −13→13
*T*_min_ = 0.952, *T*_max_ = 0.952	*k* = −23→23
11840 measured reflections	*l* = −16→16

### Refinement

**Table:**

Refinement on *F*^2^	Primary atom site location: structure-invariant direct methods
Least-squares matrix: full	Secondary atom site location: difference Fourier map
*R*[*F*^2^ > 2σ(*F*^2^)] = 0.050	Hydrogen site location: inferred from neighbouring sites
*wR*(*F*^2^) = 0.122	H atoms treated by a mixture of independent and constrained refinement
*S* = 0.99	*w* = 1/[σ^2^(*F*_o_^2^) + (0.0569*P*)^2^] where *P* = (*F*_o_^2^ + 2*F*_c_^2^)/3
2807 reflections	(Δ/σ)_max_ < 0.001
221 parameters	Δρ_max_ = 0.41 e Å^−^^3^
0 restraints	Δρ_min_ = −0.43 e Å^−^^3^

### Special details

**Table:**

Geometry. All esds (except the esd in the dihedral angle between two l.s. planes) are estimated using the full covariance matrix. The cell esds are taken into account individually in the estimation of esds in distances, angles and torsion angles; correlations between esds in cell parameters are only used when they are defined by crystal symmetry. An approximate (isotropic) treatment of cell esds is used for estimating esds involving l.s. planes.
Refinement. Refinement of F^2^ against ALL reflections. The weighted R-factor wR and goodness of fit S are based on F^2^, conventional R-factors R are based on F, with F set to zero for negative F^2^. The threshold expression of F^2^ > 2sigma(F^2^) is used only for calculating R-factors(gt) etc. and is not relevant to the choice of reflections for refinement. R-factors based on F^2^ are statistically about twice as large as those based on F, and R- factors based on ALL data will be even larger.

### Fractional atomic coordinates and isotropic or equivalent isotropic displacement parameters (Å^2^)

**Table:**

	*x*	*y*	*z*	*U*_iso_*/*U*_eq_	
C1	0.6876 (3)	0.58948 (19)	0.7714 (2)	0.0396 (9)	
C2	0.8061 (3)	0.6268 (2)	0.7974 (3)	0.0527 (10)	
H2A	0.8067	0.6572	0.8505	0.063*	
H2B	0.8701	0.5946	0.8214	0.063*	
C3	0.8350 (3)	0.6664 (2)	0.7174 (3)	0.0460 (9)	
C4	0.8408 (3)	0.7344 (2)	0.7213 (3)	0.0585 (11)	
H4	0.8252	0.7564	0.7738	0.070*	
C5	0.8696 (4)	0.7721 (2)	0.6480 (4)	0.0679 (13)	
H5	0.8750	0.8184	0.6533	0.081*	
C6	0.8898 (4)	0.7412 (2)	0.5694 (3)	0.0661 (13)	
H6	0.9074	0.7667	0.5206	0.079*	
C7	0.8844 (3)	0.6714 (2)	0.5609 (3)	0.0507 (10)	
C8	0.8573 (3)	0.63328 (19)	0.6355 (3)	0.0433 (9)	
C9	0.8534 (3)	0.5631 (2)	0.6255 (3)	0.0525 (11)	
H9	0.8374	0.5371	0.6744	0.063*	
C10	0.8724 (4)	0.5324 (2)	0.5464 (4)	0.0665 (13)	
H10	0.8690	0.4860	0.5416	0.080*	
C11	0.8969 (4)	0.5702 (3)	0.4727 (4)	0.0741 (14)	
H11	0.9087	0.5489	0.4182	0.089*	
C12	0.9039 (3)	0.6382 (3)	0.4795 (3)	0.0666 (13)	
H12	0.9216	0.6629	0.4301	0.080*	
C13	0.4383 (3)	0.44910 (18)	0.5645 (2)	0.0421 (9)	
H13	0.3634	0.4697	0.5500	0.050*	
C14	0.5879 (3)	0.38798 (17)	0.5476 (3)	0.0428 (9)	
C15	0.6185 (3)	0.41847 (17)	0.6388 (2)	0.0370 (8)	
C16	0.7297 (3)	0.40796 (19)	0.7041 (3)	0.0515 (10)	
H16	0.7507	0.4278	0.7653	0.062*	
C17	0.8083 (4)	0.3663 (2)	0.6737 (4)	0.0638 (12)	
H17	0.8842	0.3582	0.7154	0.077*	
C18	0.7766 (4)	0.3365 (2)	0.5830 (4)	0.0684 (13)	
H18	0.8319	0.3087	0.5658	0.082*	
C19	0.6675 (4)	0.34618 (19)	0.5176 (3)	0.0556 (11)	
H19	0.6472	0.3261	0.4565	0.067*	
N1	0.5211 (2)	0.45750 (13)	0.64759 (19)	0.0380 (7)	
N2	0.4725 (3)	0.40802 (14)	0.5028 (2)	0.0434 (8)	
H2	0.4300	0.3964	0.4458	0.052*	
Ni1	0.5000	0.52246 (3)	0.7500	0.0369 (2)	
O2	0.6755 (2)	0.54170 (12)	0.82476 (17)	0.0469 (7)	
O4	0.6028 (2)	0.60642 (12)	0.69919 (17)	0.0441 (6)	
O1W	0.5000	0.7341 (3)	0.7500	0.163 (4)	
H1A	0.541 (8)	0.711 (4)	0.716 (8)	0.245*	

### Atomic displacement parameters (Å^2^)

**Table:**

	*U*^11^	*U*^22^	*U*^33^	*U*^12^	*U*^13^	*U*^23^
C1	0.038 (2)	0.052 (2)	0.032 (2)	−0.0045 (18)	0.0159 (17)	−0.0054 (18)
C2	0.042 (2)	0.069 (3)	0.048 (2)	−0.011 (2)	0.0140 (18)	0.000 (2)
C3	0.028 (2)	0.054 (3)	0.056 (2)	−0.0035 (18)	0.0110 (17)	0.001 (2)
C4	0.047 (3)	0.058 (3)	0.073 (3)	−0.003 (2)	0.019 (2)	−0.010 (2)
C5	0.057 (3)	0.047 (3)	0.097 (4)	−0.007 (2)	0.017 (3)	0.012 (3)
C6	0.052 (3)	0.074 (4)	0.074 (3)	−0.004 (2)	0.019 (2)	0.022 (3)
C7	0.032 (2)	0.066 (3)	0.054 (3)	0.001 (2)	0.0117 (18)	0.007 (2)
C8	0.0237 (19)	0.048 (3)	0.057 (2)	0.0011 (17)	0.0101 (17)	0.006 (2)
C9	0.034 (2)	0.057 (3)	0.068 (3)	−0.0009 (19)	0.017 (2)	0.000 (2)
C10	0.042 (3)	0.070 (3)	0.086 (3)	0.011 (2)	0.015 (2)	−0.020 (3)
C11	0.044 (3)	0.109 (4)	0.069 (3)	0.021 (3)	0.014 (2)	−0.021 (3)
C12	0.041 (3)	0.107 (4)	0.055 (3)	0.009 (3)	0.018 (2)	0.009 (3)
C13	0.041 (2)	0.046 (2)	0.042 (2)	−0.0002 (18)	0.0162 (18)	0.0009 (18)
C14	0.049 (2)	0.040 (2)	0.046 (2)	−0.0024 (19)	0.0236 (19)	0.0021 (18)
C15	0.036 (2)	0.038 (2)	0.040 (2)	−0.0002 (17)	0.0171 (17)	0.0012 (17)
C16	0.045 (2)	0.052 (3)	0.058 (3)	0.003 (2)	0.016 (2)	0.002 (2)
C17	0.043 (3)	0.060 (3)	0.088 (3)	0.008 (2)	0.016 (2)	0.004 (3)
C18	0.064 (3)	0.060 (3)	0.094 (4)	0.015 (2)	0.043 (3)	−0.004 (3)
C19	0.064 (3)	0.052 (3)	0.061 (3)	0.000 (2)	0.035 (2)	−0.006 (2)
N1	0.0346 (17)	0.0430 (18)	0.0379 (16)	0.0012 (14)	0.0127 (14)	0.0027 (14)
N2	0.045 (2)	0.0480 (19)	0.0362 (16)	−0.0053 (16)	0.0095 (15)	−0.0029 (15)
Ni1	0.0321 (4)	0.0448 (4)	0.0365 (4)	0.000	0.0142 (3)	0.000
O2	0.0376 (15)	0.0565 (17)	0.0476 (15)	−0.0063 (12)	0.0132 (12)	0.0065 (13)
O4	0.0357 (15)	0.0578 (17)	0.0412 (14)	−0.0039 (12)	0.0143 (12)	−0.0008 (12)
O1W	0.097 (5)	0.066 (4)	0.320 (13)	0.000	0.046 (6)	0.000

### Geometric parameters (Å, º)

**Table:**

C1—O2	1.254 (4)	C13—N1	1.316 (4)
C1—O4	1.260 (4)	C13—N2	1.341 (4)
C1—C2	1.516 (5)	C13—H13	0.9300
C1—Ni1	2.498 (4)	C14—N2	1.374 (4)
C2—C3	1.502 (5)	C14—C15	1.394 (5)
C2—H2A	0.9700	C14—C19	1.395 (5)
C2—H2B	0.9700	C15—C16	1.383 (5)
C3—C4	1.361 (5)	C15—N1	1.405 (4)
C3—C8	1.429 (5)	C16—C17	1.389 (5)
C4—C5	1.403 (6)	C16—H16	0.9300
C4—H4	0.9300	C17—C18	1.382 (6)
C5—C6	1.356 (6)	C17—H17	0.9300
C5—H5	0.9300	C18—C19	1.364 (6)
C6—C7	1.401 (6)	C18—H18	0.9300
C6—H6	0.9300	C19—H19	0.9300
C7—C12	1.410 (5)	N1—Ni1	2.021 (3)
C7—C8	1.413 (5)	N2—H2	0.8600
C8—C9	1.410 (5)	Ni1—N1^i^	2.021 (3)
C9—C10	1.355 (5)	Ni1—O2	2.055 (2)
C9—H9	0.9300	Ni1—O2^i^	2.055 (2)
C10—C11	1.386 (6)	Ni1—O4	2.287 (2)
C10—H10	0.9300	Ni1—O4^i^	2.288 (2)
C11—C12	1.364 (6)	Ni1—C1^i^	2.498 (4)
C11—H11	0.9300	O1W—H1A	0.90 (8)
C12—H12	0.9300		
			
O2—C1—O4	120.7 (3)	C14—C15—N1	108.8 (3)
O2—C1—C2	118.1 (3)	C15—C16—C17	116.6 (4)
O4—C1—C2	121.2 (3)	C15—C16—H16	121.7
O2—C1—Ni1	55.05 (17)	C17—C16—H16	121.7
O4—C1—Ni1	65.66 (19)	C18—C17—C16	121.6 (4)
C2—C1—Ni1	172.8 (3)	C18—C17—H17	119.2
C3—C2—C1	116.6 (3)	C16—C17—H17	119.2
C3—C2—H2A	108.1	C19—C18—C17	122.7 (4)
C1—C2—H2A	108.1	C19—C18—H18	118.7
C3—C2—H2B	108.1	C17—C18—H18	118.7
C1—C2—H2B	108.1	C18—C19—C14	116.1 (4)
H2A—C2—H2B	107.3	C18—C19—H19	122.0
C4—C3—C8	118.6 (4)	C14—C19—H19	122.0
C4—C3—C2	120.9 (4)	C13—N1—C15	104.6 (3)
C8—C3—C2	120.5 (4)	C13—N1—Ni1	122.2 (2)
C3—C4—C5	121.7 (4)	C15—N1—Ni1	132.9 (2)
C3—C4—H4	119.2	C13—N2—C14	107.3 (3)
C5—C4—H4	119.2	C13—N2—H2	126.4
C6—C5—C4	120.2 (4)	C14—N2—H2	126.4
C6—C5—H5	119.9	N1—Ni1—N1^i^	100.03 (15)
C4—C5—H5	119.9	N1—Ni1—O2	101.45 (10)
C5—C6—C7	120.8 (4)	N1^i^—Ni1—O2	92.41 (10)
C5—C6—H6	119.6	N1—Ni1—O2^i^	92.41 (10)
C7—C6—H6	119.6	N1^i^—Ni1—O2^i^	101.45 (10)
C6—C7—C12	121.8 (4)	O2—Ni1—O2^i^	158.42 (14)
C6—C7—C8	119.1 (4)	N1—Ni1—O4	93.73 (10)
C12—C7—C8	119.1 (4)	N1^i^—Ni1—O4	151.40 (10)
C9—C8—C7	117.9 (4)	O2—Ni1—O4	60.13 (9)
C9—C8—C3	122.4 (4)	O2^i^—Ni1—O4	102.90 (9)
C7—C8—C3	119.6 (4)	N1—Ni1—O4^i^	151.40 (10)
C10—C9—C8	121.7 (4)	N1^i^—Ni1—O4^i^	93.73 (10)
C10—C9—H9	119.1	O2—Ni1—O4^i^	102.90 (9)
C8—C9—H9	119.1	O2^i^—Ni1—O4^i^	60.13 (9)
C9—C10—C11	120.1 (4)	O4—Ni1—O4^i^	85.60 (12)
C9—C10—H10	120.0	N1—Ni1—C1	99.19 (11)
C11—C10—H10	120.0	N1^i^—Ni1—C1	121.98 (11)
C12—C11—C10	120.6 (4)	O2—Ni1—C1	30.01 (10)
C12—C11—H11	119.7	O2^i^—Ni1—C1	131.69 (12)
C10—C11—H11	119.7	O4—Ni1—C1	30.12 (10)
C11—C12—C7	120.6 (4)	O4^i^—Ni1—C1	94.46 (11)
C11—C12—H12	119.7	N1—Ni1—C1^i^	121.98 (11)
C7—C12—H12	119.7	N1^i^—Ni1—C1^i^	99.19 (11)
N1—C13—N2	113.5 (3)	O2—Ni1—C1^i^	131.69 (12)
N1—C13—H13	123.3	O2^i^—Ni1—C1^i^	30.01 (10)
N2—C13—H13	123.3	O4—Ni1—C1^i^	94.46 (11)
N2—C14—C15	105.9 (3)	O4^i^—Ni1—C1^i^	30.12 (10)
N2—C14—C19	132.2 (4)	C1—Ni1—C1^i^	115.13 (18)
C15—C14—C19	122.0 (4)	C1—O2—Ni1	94.9 (2)
C16—C15—C14	121.1 (3)	C1—O4—Ni1	84.2 (2)
C16—C15—N1	130.1 (3)		
			
O2—C1—C2—C3	159.6 (3)	C13—N1—Ni1—O2	−156.1 (3)
O4—C1—C2—C3	−21.6 (5)	C15—N1—Ni1—O2	16.7 (3)
Ni1—C1—C2—C3	177 (2)	C13—N1—Ni1—O2^i^	7.2 (3)
C1—C2—C3—C4	113.3 (4)	C15—N1—Ni1—O2^i^	−180.0 (3)
C1—C2—C3—C8	−66.9 (5)	C13—N1—Ni1—O4	−95.9 (3)
C8—C3—C4—C5	−0.9 (6)	C15—N1—Ni1—O4	76.9 (3)
C2—C3—C4—C5	178.9 (4)	C13—N1—Ni1—O4^i^	−8.2 (4)
C3—C4—C5—C6	1.7 (6)	C15—N1—Ni1—O4^i^	164.6 (2)
C4—C5—C6—C7	−1.2 (6)	C13—N1—Ni1—C1	−125.7 (3)
C5—C6—C7—C12	179.4 (4)	C15—N1—Ni1—C1	47.1 (3)
C5—C6—C7—C8	0.1 (6)	C13—N1—Ni1—C1^i^	1.8 (3)
C6—C7—C8—C9	−179.5 (3)	C15—N1—Ni1—C1^i^	174.6 (3)
C12—C7—C8—C9	1.2 (5)	O2—C1—Ni1—N1	−97.0 (2)
C6—C7—C8—C3	0.6 (5)	O4—C1—Ni1—N1	81.5 (2)
C12—C7—C8—C3	−178.7 (3)	C2—C1—Ni1—N1	−116 (2)
C4—C3—C8—C9	179.9 (3)	O2—C1—Ni1—N1^i^	11.0 (2)
C2—C3—C8—C9	0.1 (5)	O4—C1—Ni1—N1^i^	−170.49 (17)
C4—C3—C8—C7	−0.2 (5)	C2—C1—Ni1—N1^i^	−8 (2)
C2—C3—C8—C7	−180.0 (3)	O4—C1—Ni1—O2	178.5 (3)
C7—C8—C9—C10	−1.3 (5)	C2—C1—Ni1—O2	−19 (2)
C3—C8—C9—C10	178.6 (4)	O2—C1—Ni1—O2^i^	161.39 (15)
C8—C9—C10—C11	0.2 (6)	O4—C1—Ni1—O2^i^	−20.1 (3)
C9—C10—C11—C12	0.9 (6)	C2—C1—Ni1—O2^i^	142 (2)
C10—C11—C12—C7	−1.0 (6)	O2—C1—Ni1—O4	−178.5 (3)
C6—C7—C12—C11	−179.4 (4)	C2—C1—Ni1—O4	162 (2)
C8—C7—C12—C11	−0.1 (6)	O2—C1—Ni1—O4^i^	108.2 (2)
N2—C14—C15—C16	179.0 (3)	O4—C1—Ni1—O4^i^	−73.3 (2)
C19—C14—C15—C16	−0.5 (5)	C2—C1—Ni1—O4^i^	89 (2)
N2—C14—C15—N1	−0.7 (4)	O2—C1—Ni1—C1^i^	131.1 (2)
C19—C14—C15—N1	179.7 (3)	O4—C1—Ni1—C1^i^	−50.43 (17)
C14—C15—C16—C17	0.5 (5)	C2—C1—Ni1—C1^i^	112 (2)
N1—C15—C16—C17	−179.8 (3)	O4—C1—O2—Ni1	−1.6 (4)
C15—C16—C17—C18	−0.4 (6)	C2—C1—O2—Ni1	177.3 (3)
C16—C17—C18—C19	0.4 (7)	N1—Ni1—O2—C1	88.6 (2)
C17—C18—C19—C14	−0.4 (6)	N1^i^—Ni1—O2—C1	−170.7 (2)
N2—C14—C19—C18	−179.0 (4)	O2^i^—Ni1—O2—C1	−40.39 (19)
C15—C14—C19—C18	0.5 (6)	O4—Ni1—O2—C1	0.86 (19)
N2—C13—N1—C15	0.3 (4)	O4^i^—Ni1—O2—C1	−76.3 (2)
N2—C13—N1—Ni1	174.9 (2)	C1^i^—Ni1—O2—C1	−66.1 (3)
C16—C15—N1—C13	−179.5 (4)	O2—C1—O4—Ni1	1.4 (3)
C14—C15—N1—C13	0.3 (4)	C2—C1—O4—Ni1	−177.4 (3)
C16—C15—N1—Ni1	6.8 (6)	N1—Ni1—O4—C1	−101.9 (2)
C14—C15—N1—Ni1	−173.4 (2)	N1^i^—Ni1—O4—C1	17.0 (3)
N1—C13—N2—C14	−0.8 (4)	O2—Ni1—O4—C1	−0.86 (19)
C15—C14—N2—C13	0.9 (4)	O2^i^—Ni1—O4—C1	164.7 (2)
C19—C14—N2—C13	−179.5 (4)	O4^i^—Ni1—O4—C1	106.7 (2)
C13—N1—Ni1—N1^i^	109.3 (3)	C1^i^—Ni1—O4—C1	135.57 (19)
C15—N1—Ni1—N1^i^	−77.9 (3)		

Symmetry code: (i) −*x*+1, *y*, −*z*+3/2.

### Hydrogen-bond geometry (Å, º)

**Table:**

*D*—H···*A*	*D*—H	H···*A*	*D*···*A*	*D*—H···*A*
O1*W*—H1*A*···O4	0.90 (8)	2.24 (8)	2.988 (6)	141 (8)
N2—H2···O4^ii^	0.86	2.00	2.791 (4)	152

Symmetry code: (ii) −*x*+1, −*y*+1, −*z*+1.
